# Differential Proteomic Analysis of the Pancreas of Diabetic *db*/*db* Mice Reveals the Proteins Involved in the Development of Complications of Diabetes Mellitus

**DOI:** 10.3390/ijms15069579

**Published:** 2014-05-30

**Authors:** Victoriano Pérez-Vázquez, Juan M. Guzmán-Flores, Daniela Mares-Álvarez, Magdalena Hernández-Ortiz, Maciste H. Macías-Cervantes, Joel Ramírez-Emiliano, Sergio Encarnación-Guevara

**Affiliations:** 1 Depto. de Ciencias Médicas, División de Ciencias de la Salud, Campus León, Universidad de Guanajuato, León, Guanajuato 37320, Mexico; E-Mails: manuguz@yahoo.com.mx (J.M.G.-F.); danymares7@hotmail.com (D.M.-A.); macistehabacuc@yahoo.com.mx (M.H.M.-C.); joelre@ugto.mx (J.R.-E.); 2 Centro de Ciencias Genómicas, Universidad Nacional Autónoma de México, Cuernavaca, Morelos 62210, Mexico; E-Mails: magdini@gmail.com (M.H.-O.); encarnac@ccg.unam.mx (S.E.-G.)

**Keywords:** *db*/*db* mice, diabetes mellitus, obesity, pancreas, proteomics, interactome

## Abstract

Type 2 diabetes mellitus is characterized by hyperglycemia and insulin-resistance. Diabetes results from pancreatic inability to secrete the insulin needed to overcome this resistance. We analyzed the protein profile from the pancreas of ten-week old diabetic *db*/*db* and wild type mice through proteomics. Pancreatic proteins were separated in two-dimensional polyacrylamide gel electrophoresis (2D-PAGE) and significant changes in *db*/*db* mice respect to wild type mice were observed in 27 proteins. Twenty five proteins were identified by matrix-assisted laser desorption/ionization (MALDI) time-of-flight (TOF) and their interactions were analyzed using search tool for the retrieval of interacting genes/proteins (STRING) and database for annotation, visualization and integrated discovery (DAVID). Some of these proteins were Pancreatic α-amylase, Cytochrome b5, Lithostathine-1, Lithostathine-2, Chymotrypsinogen B, Peroxiredoxin-4, Aspartyl aminopeptidase, Endoplasmin, and others, which are involved in the metabolism of carbohydrates and proteins, as well as in oxidative stress, and inflammation. Remarkably, these are mostly endoplasmic reticulum proteins related to peptidase activity, *i.e.*, they are involved in proteolysis, glucose catabolism and in the tumor necrosis factor-mediated signaling pathway. These results suggest mechanisms for insulin resistance, and the chronic inflammatory state observed in diabetes.

## 1. Introduction

Pancreatic endocrine and exocrine cells synthesize and secrete hormones and enzymes required for nutritional balance. The endocrine portion of the pancreas regulates blood glucose levels through the alternative secretion of insulin and glucagon by beta and alpha cells, respectively. Exocrine pancreatic cells secrete several digestive enzymes. Type 2 diabetes mellitus (T2DM) represents a highly complex and heterogeneous disease that is influenced by both genetic and environmental factors. Insulin resistance is a primary defect in T2DM, where the uptake of glucose into muscle is impaired. When pancreatic cells become incapable to compensate for this insulin resistance by increasing insulin secretion, T2DM ensues [1,2]. Proposed mechanisms to explain insulin resistance and islet β-cell dysfunction in T2DM are oxidative stress, endoplasmic reticulum (ER) stress, amyloid deposition in the pancreas, ectopic lipid deposition in the muscle, liver and pancreas, and general lipotoxicity and glucotoxicity. Obesity can cause all of these phenomena, although it has been difficult to determine which mechanism is the most important each tissue and in each T2DM model or individual [2,3]. Animal models have contributed greatly to our understanding of T2DM and have helped to develop new therapeutics. One commonly used model is *db*/*db* mice that exhibit a mutation in the gene encoding the leptin receptor; this T2DM model has been well characterized and it has been observed that disease develops similarly to humans, including hyperphagia, hyperglycemia, insulin resistance and obesity [4].

There are several powerful techniques to study disease-borne alterations. Among these are, microarray, Genome-Wide Association Studies and proteomics. We used proteomics, which allows for comparison of protein profiles in normal and diseased tissues or cells and may lead to identify proteins or signaling pathways involved in pathogenesis [5]. Sanchez *et al*. [6] published an update to Swiss two-dimensional polyacrylamide gel electrophoresis (SWISS-2D PAGE) database with 2-D gels from mouse (C57BL76J) pancreatic islets. In addition, Qiu *et al*. [7] identified pancreatic proteins differentially expressed in normal mice and those with diet-induced T2DM. However, only 4 proteins were identified with change expression in diabetic mice. Although other studies exists, they were performed in pancreatic islets. Our goal was to obtain a detailed understanding of the changes in pancreatic proteins that associate with obesity/diabetes in a mouse model identifying differentially expressed proteins.

## 2. Results and Discussion

Body weights were significantly higher in *db*/*db* mice compared with wild type (WT) mice (42.23 ± 2.38 and 29.24 ± 0.63 g, respectively; *p* < 0.001), similar differences were also found in the blood glucose levels (547.67 ± 2.84 and 229.00 ± 17.21 mg/dL, respectively; *p* < 0.001), indicating that the *db*/*db* mice are obese and diabetic as reported by others [8].

**Figure 1:**
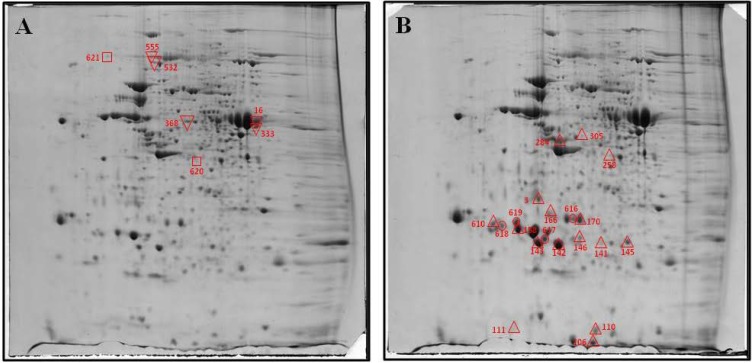
Two-dimensional electrophoresis (2-DE) image of the pancreas of control (**A**) and *db*/*db* (**B**) mouse. Proteins were separated by isoelectric focusing (IEF) in the first dimension, pH 3–10 enriched in range from 4–8, then by size in the second dimension. Spots found with differential expression are marked in the gels. **A**: □, proteins presents only in control mice and, ▽, diminished proteins in *db/db* mice; **B**: △, increased proteins in *db*/*db* mice and, O spots found only in *db*/*db* mice.

Two-dimensional PAGE analysis highlighted several differences in *db*/*db* mice *versus* matched wild type mice. As shown in the representative images (Figure 1), about 620 spots were detected in each image. The protein spot distribution of normal *versus* diabetic preparations did not show extensive differences, but densitometric analysis revealed moderate alterations in several pancreatic proteins. From the 620 resolved protein spots, 27 exhibited a difference of 1.5-fold or greater from *db*/*db* mice to WT mice. The locations of these 27 spots on a 2-D gel are marked and numbered (Figure 1).

The pancreatic proteins for 25 spots that exhibited a significantly altered expression level in *db*/*db* mice were identified (Table 1). The table summarizes proteins separated in the pH 3–10 enriched in 4–8 range with percentage sequence coverage, Mascot score, the relative molecular mass, pI-value, protein accession number and fold-change of individual pancreas proteins affected by the diabetic phenotype. Of these 25 protein spots, 15 increased, and 4 were found only in *db*/*db* mice, 4 diminished in *db*/*db* mice and 2 exist only in control mice. Note that some spots were identified as the same protein.

In T2DM there are defects in pancreatic insulin secretion and/or effects [1,2]. Thus, studying the pancreatic protein alterations that occur T2DM is of particular interest.

**Table 1:** Proteins identified differentially expressed in the mouse pancreas *db*/*db*.

Spot number	Protein	Gene name	UniProt accession number	Molecular mass (kD)	Peptide matched	Mascot score	Sequence coverage (%)	Fold change
111	Cytochrome b5	*Cyb5a*	P56395	15.232	6	84	49	+2.39
106	Lithostathine 1	*Reg1*	P43137	18.906	9	132	55	+2.38
110	Lithostathine-2	*Reg2*	Q08731	19.793	7	109	52	+2.51
610	Prss3 protein	*Prss3*	Q4G0C2	26.643	7	74	24	+2.8
618	Chymotrypsinogen B	*Ctrb1*	Q9CR35	28.585	8	89	42	^a^
619	Trypsinogen 11	*Trypsinogen*	Q792Z0	268.88	7	80	31	^a^
158	Chymotrypsinogen B precursor	*Ctrb1*	Q9CR35	28.585	5	78	31	+1.55
143	Trypsin 5 precursor	*Try5*	Q9QUK9	27.112	9	80	31	+2.41
142	Trypsin 5 precursor	*Try5*	Q9QUK9	27.112	10	111	31	+1.71
146	6-Phosphogluconolactonase	*Pgls*	Q9CQ60	27.522	8	112	43	+2.07
141	Peroxiredoxin-4 precursor	*Prdx4*	O08807	31.317	9	131	43	+1.6
145	Phosphoglycerate mutase 1	*Pgam1*	Q9DBJ1	28.956	11	131	55	+2.2
166	Ela3 protein, partial	*Ela3*	A2A9U8	28.321	7	83	29	+2.25
616	Ela3 protein, partial	*Ela3*	A2A9U8	28.321	7	102	47	^a^
170	Ela3 protein, partial	*Ela3*	A2A9U8	28.321	10	129	68	+1.63
3	Kallikrein	*Klk1*	P15947	29.495	8	97	37	+1.68
258	Protein MYG1, mitochondrial	*Myg1*	Q9JK81	43.095	7	74	24	+1.81
284	Pancreatic Carboxypeptidase B1 precursor	*Cpb1*	B2RS76	48.041	15	204	48	+2.25
305	Carboxypeptidase A2, pancreatic, isoform CRA_b	*Cpa2*	Q504N0	37.185	10	99	26	+1.98
620	Aspartyl aminopeptidase	*Dnpep*	Q9Z2W0	52.744	9	69	17	^b^
621	Endoplasmin	*Hsp90b1*	P08113	92.703	13	112	13	^b^
555	Endoplasmin	*Hsp90b1*	P08113	92.998	20	174	25	−2.04
532	Transitional endoplasmic reticulum ATPase	*Vcp*	Q01853	89.950	15	113	16	−1.5
368	Keratin, type II cytoskeletal 8	*Krt8*	P11679	54.531	24	205	47	−1.99
16	Pancreatic alpha-amylase	*Amy2a*	P00688	57.966	8	89	21	−1.83

^a^, Protein spots 616, 618 and 619 exist only in *db*/*db* mice; ^b^, Protein spots 620 and 621 exist only in control mice.

The proteins related to carbohydrate metabolism and found altered in the *db*/*db* mouse were Pancreatic alpha-amylase (AMY2A), Keratin type II cytoskeletal 8 (KRT8) and Phosphoglycerate mutase 1 (PGAM1). Decreased expression of AMY2A2 previously has been reported in islets of Langerhans cultured with high glucose concentrations [9], as well as in hyperlipidemic rat pancreas [10,11]. In this regard, as amylase is synthesized and stored in pancreatic acinar cells, damage of these cells might result in the partial loss of pancreatic amylase. Otherwise, lipid accumulation and decreased carbohydrate in diets probably suppresses the need for pancreatic amylase expression [10,11]. KRT8 together with KRT19 collaborate with KRT18 to regulate the metabolism of glucose through modulation of mitochondrial hexokinase [12]. KRT8 has been found up-regulated in cultured islets [9], this contrasts with our results. This may be explained by the model, i.e. we use the full pancreas from genetically modified *db*/*db* mice. PGAM1 is an enzyme involved in the second phase of glycolysis, although there are some discrepancies as to whether this metabolic pathway is increased or decreased at high concentrations of glucose, it is clear that in T2DM this pathway is disturbed [13,14,15]. At least in type 1 diabetes, it has been demonstrated that multiple alterations in the expression of proteins involved in oxidative stress, aerobic and anaerobic glycolysis and intracellular signaling in human skin were reversed after kidney-pancreas transplant [16]. Therefore, pancreas is a key organ in diabetes complications in whole organism.

As expected from data in the literature [17,18], a large number of proteolytic pathways-related proteins were differentially expressed. Such proteins were Aspartyl aminopeptidase (DNPEP), Chymotrypsinogen B (CTRB1), Prss3 protein (PRSS3), Elastase 3 (ELA3), Kallikrein (KLK1), Carboxypeptidase B1 (CPB1) and Carboxypeptidase A2 (CPA2). Previously, a polymorphism in the gene *Ctrb1* has been associated with diabetes susceptibility and treatment via the incretin pathway [19]. The PRSS3 protein, also known as trypsinogen IV, trypsinogen 9 or trypsin IV was secreted in large quantities in obese Zucker rats [20], besides that it has been proposed that this protein induces inflammation [21], a process related to T2DM [3]. Moreover this molecule is differentially expressed in mice null for the α2A adrenergic receptor, which is characterized by attenuated glucose-stimulated insulin release from pancreatic β cells [22]. ELA3 is part of a subfamily of serine proteases. It has been reported that this protein is expressed in pancreatic carcinoma cells [23] and in patients with diabetes with poor glycemic control it was associated with a higher risk of presenting a low ELA1 level [24]. KLK1 is a protein that has previously been reported with high levels in patients with T2DM and its administration attenuated insulin resistance [25,26].

In regard to carboxypeptidases, we found over-expression of CPA2 and CPB1 in the mouse *db*/*db*. The latter has been linked to activation of insulin, and both carboxypeptidases were over-expressed in α2A adrenergic receptor knockout mice. The increase in an enzyme involved in insulin processing may represent a compensatory effort against the loss of tonic suppression of insulin release [22].

In T2DM, ER stress, oxidative stress, amyloid deposition, lipotoxicity and glucotoxicity seem to combine to promote insulin resistance and islet β-cell dysfunction [3]. We found two proteins related to ER stress, endoplasmin and Valosin-containing protein (VCP), and two with oxidative stress, Peroxiredoxin-4 (PRDX4) and 6-Phosphogluconolactonase (6PGL). Endoplasmin is a molecular chaperone that functions in the processing and transport of secreted proteins: This protein has been linked to stress in pancreatic β cells from Akita mouse [27] and is overexpressed in pancreatic cells cultured in the presence of high glucose [9,28]. This is in contrast with our findings; on the other hand, this same molecule decreased in *db*/*db* mice hippocampus [29]. In addition, we identified the transitional endoplasmic reticulum ATPase or, VCP protein, which is necessary for the fragmentation of Golgi stacks during mitosis and their reassembly after mitosis. Low levels of VCP are reported during ER stress [30]. PRDX4 also increased in diabetic *db*/*db* mice; this protein is probably involved in cellular redox regulation and in activation of nuclear factor kkapa B (NF-*κ*B) in the cytosol. Genome-wide association studies report that patients with T2DM have high expression levels of pancreatic PRDX4 [31]. Moreover, the pancreatic proteome profile from streptozotocin-induced diabetic rats shows that, PRDX4 was down-regulated [32]. Interestingly, overexpression of PRDX4 protects against streptozotocin-induced diabetes by suppressing oxidative stress and cytokines in the transgenic mice [33]. In any case is very likely that PRDX4 plays a key role in controlling T2DM, as it exhibits high protective activity against oxidative [3,34]. In turn, regulation of oxidative stress depends on some antioxidants, such as NADPH that is produced in the pentose phosphate pathway (PPP), 6GPL is an enzyme involved in this pathway. Additionally, skeletal muscle metabolites from PPP increase in obese mice [35], in rat beta cell line INS-1E and in human islets [36]. It has been reported that Glucose-6-phosphate dehydrogenase (G6PD), the rate-limiting enzyme in the PPP, increased both in Zucker obese fa/fa rats and in obese type 2 diabetics [37,38], but its increased activity and concentration of metabolites are reversed by adiponectin or post-laparoscopic Roux-en-Y gastric bypass [35,38]; this is the first study reporting over expression of 6GLD in T2DM.

Systemic oxidative stress and ER stress are produced by obesity and aging and eventually lead to the development of T2DM [39,40,41]. In this study, we report two proteins related to these factors. Cytochrome b5 (CYB5) is an electron transfer component in a number of oxidative reactions, including the anabolic metabolism of fats and steroids. CYB5 has previously been associated with obesity in New Zealand obese mouse [42], but not diabetes.

The mechanisms mentioned above to explain insulin resistance and islet β-cell dysfunction in T2DM, obesity and ageing are thought to either induce an inflammatory response or to be exacerbated by or associated with-inflammation [3]. This process involves different subsets of the immune system cells such as macrophages, neutrophils, eosinophils, Th1, Th2, T regulatory and Th17, as well as the cytokines Tumor necrosis factor alpha (TNF-α), IL-6, IL-10, among others [3,43,44]. Here some inflammation-related proteins were detected in the *db*/*db* mouse. Lithostathine 1 (REG1) and REG2 increased expression in the pancreas of diabetic *db*/*db* mice as compared wild type mice. It has been reported that REG1 and REG2 induce neutrophil activation [45] so that they might contribute to the diabetic inflammatory process. In another study using 2-D gel high expression of REG1 and REG2 was observed, probably reflecting an effort to stimulate the proliferation of pancreatic beta cells enhancing insulin secretion [7]. Protein MYG1, mitochondrial (MYG1) was also overexpressed. This recently described protein is probably involved in early development and in immune-related processes [46].

**Figure 2:**
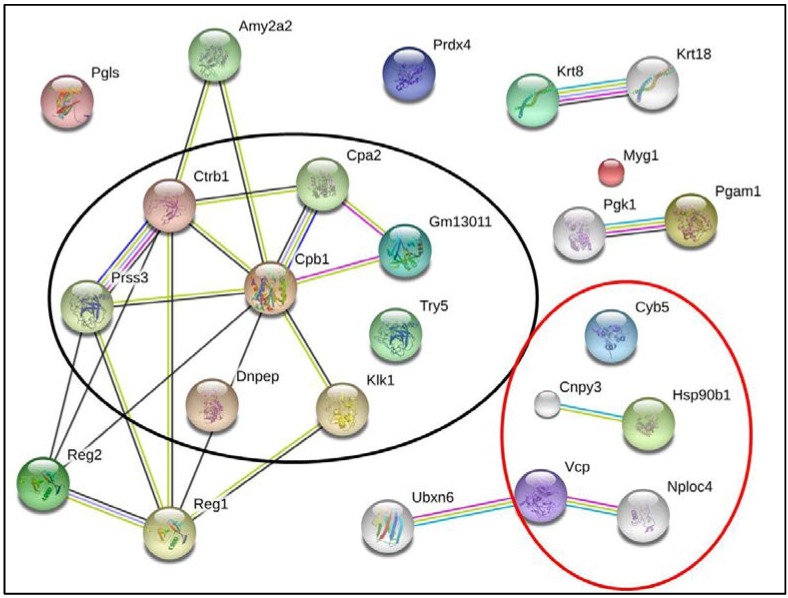
Bioinformatics analysis by Search Tool for the Retrieval of Interacting Genes/Proteins. The list of the identified protein was subjected to String (v. 9.05) analysis to reveal functional interactions between the deregulated proteins. Each node represents a protein, and each edge represents an interaction. The original graphic output was modified to fit the proteins, according to their classification under the gene ontology descriptors “Cellular Component”: endoplasmic reticulum (confined within the red circle) and “Molecular Function”: peptidases activity (confined within the black circle) as revealed by the Database for Annotation, Visualization and Integrated Discovery (DAVID) (v. 6.7) annotation system.

**Figure 3:**
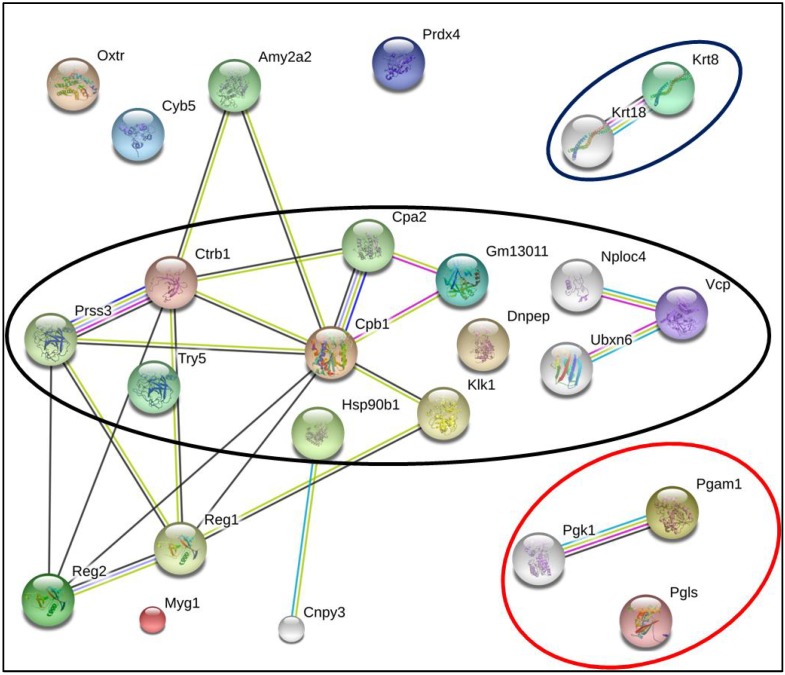
Bioinformatics analysis by String. The list of the identified proteins was subjected to String (v. 9.05) analysis to reveal functional interactions between the deregulated proteins. Each node represents a protein, and each edge represents an interaction. The original graphic output was modified to fit the proteins, according to their classification under the gene ontology descriptor “Biological Process”: proteolysis (confined within the red circle), glucose catabolic process (confined within the black circle) and tumor necrosis factor-mediated signaling pathway (confined within the blue circle) as revealed by the DAVID (v. 6.7) annotation system.

As shown in Figure 2 and Figure 3, in order to find relevant proteins among the multiple identifications obtained by proteomic analysis, we subjected the list of the 21 different proteins from Table 1 to bioinformatics analysis in the STRING database, which integrates interaction data from several bioinformatics sources and provides information about physical and functional properties, known and predicted interactions of genes and their products [47].

Five additional interacting proteins were added to provide a more comprehensive view of the interactions. Further analysis of the biological meaning of the studied proteins was carried out using the database DAVID, which analyzes gene or protein lists deriving from high-throughput experiments and systematically extracts biological meaning from them. DAVID was also used to highlight the functional annotation clustering inside the identified potential protein network [48]. As seen in Figure 2, it was found that proteins belonging to “endoplasmic reticulum” (GOTERM_CC_FAT *P* = 8, 8E-3) and having a molecular function related “peptidase activity” (GOTERM_MF_FAT *P* = 2, 3E-5). Figure 3 shows that these proteins are also involved in “proteolysis” (GOTERM_BP_FAT *P* = 2, 8E-8) and that these proteins are involved in the “tumor necrosis factor-mediated signaling pathway” (GOTERM_BP_FAT *P* = 5, 6E-3) as well as in “glucose catabolic process” (GOTERM_BP_FAT *P* = 2, 4E-3).

ER is a central organelle for the synthesis of secretory and membrane proteins along with membrane lipids and internal calcium storage. Newly synthesized proteins are modified and folded into their native structure within the ER lumen, which is tightly monitored by the ER quality control machinery. Protein synthesis in the ER is dynamically adjusted in coordination with the physiological status of cells. When the folding capacity of the ER fails to accommodate the load of unfolded proteins, ER homeostasis is perturbed to a condition referred to as “ER stress.” [49]. Conditions of insulin resistance and obesity cause hyperglycemia and hyperlipidemia, which result in glucolipotoxicity for the β-cell. Studies suggest that free fatty acid-mediated unfolded protein response signaling pathways are potentiated by high-glucose cosupplementation to β-cells as high glucose exacerbates β-cell lipotoxicity. The amplified ER stress response leads to β-cell dysfunction and apoptosis through proinsulin mRNA degradation, oxidative stress, proapoptotic signals, and mitochondrial apoptosis, eventually culminating in T2DM [50]. Therefore, the analysis of our results with DAVID and STRING databases support previous reports of the involvement of the endoplasmic reticulum in the pathophysiology of T2DM.

It has been established that proteolysis and peptidase activity are impaired in the pathophysiological process of diabetes. Specifically it has been reported that some metabolic steps of amino acid synthesis, which are related to vascular complications (methionine and arginine) exhibit a defective response to insulin in T2DM subjects [17,18]. Moreover, the recent facilitation of comprehensive metabolic profiling or metabolomics using liquid chromatography-tandem mass spectrometry (LC-MS) identified the branched-chain amino acids (BCAAs) valine, leucine and isoleucine and related metabolites to be positively related to insulin resistance. Elevated plasma concentrations of BCAAs have been reported in obese nondiabetic and nonobese insulin-resistant individuals [17,18]. Therefore, this process has some biological role in the pathophysiology of T2DM, as suggested by the analysis of our data and previous reports.

Participation of signaling pathway of TNF-α in the pathophysiology of insulin resistance and T2DM has been previously reported [3]. Inflammatory cytokines, including TNF-α, have been shown to promote insulin resistance, and altered expression of cytokines in obese adipose tissue is thought to be an important link between obesity and insulin resistance. It is also becoming clear that inflammation plays a key role in the development of β-cell dysfunction. TNF-α had been the first pro-inflammatory cytokine being associated with obesity and related insulin resistance. Further evidence supporting a key role for TNF-α in insulin resistance came from studies where they observed that mice lacking TNF-α or TNF receptors had improved insulin sensitivity in both dietary and genetic (*ob*/*ob*) models of obesity. Initial studies suggested that the defect in insulin signaling could be attributed to serine phosphorylation of insulin receptor substrate 1 at serine-307 residue by activation of jun-*N*-terminal kinase 1 (JNK1), providing a first explanation how inflammation and insulin resistance might be linked [51,52].

There is controversy on whether the catabolic hexose-processing enzymes, including glucose, decrease or increase in diabetes, as wide alterations have been observed [13,14,15].

Finally, our studies on the differential protein expression in *db*/*db* mice and their interactions, suggest that dysfunctional ER-dependent and carbohydrate metabolism processes, together with a chronic inflammation play a role in the pathogenesis of diabetes. Also, the efforts from the organism to recover are suggested by the overexpression of REG1 and REG2.

## 3. Experimental Section

### 3.1. Animals

Female and male heterozygote non-diabetic *db*/*+* mice (BKS.Cg-m +/+ Leprdb/OlaHsd; Black, Lean, were purchased from Harlan Laboratories (Mexico City, Mexico), and were back-crossed to start our colony. All animal procedures were performed in accordance with current Mexican legislation, NOM-062-ZOO-1999, and with the National Institutes of Health (NIH, Bethesda, MD, USA) Guide for the care and use of laboratory animals. Mice were kept in the animal facility of the University of Guanajuato in Leon, Mexico under standard housing conditions with water and food ad libitum and with a 12 h:12 h light/dark cycle.

### 3.2. Sample Preparation and 2D-Polyacrylamide Gel Electrophoresis

Once the mice were genotyped as described previously [53], ten-week-old males were selected and grouped in *db*/*db* and wild type mice (*n* = 3). The pancreas was removed and homogenized in a buffer containing Tris/HCl 20 mM, 10 mM EDTA, 2 mM DTT, pH 7.8 and protease inhibitor (20 mM PMSF) then stored at −70 °C until further processing. Cellular proteins were obtained by Sample Grinding Kit (GE Healthcare, Mexico City, Mexico) and resuspended in 200 μL of buffer (7 M urea, 4% CHAPS, 60 mM DTT, 2 mM TBP, 2 mM thiourea, 2% IPG). Protein concentrations were determined by 2D Quant Kit (GE Healthcare). Two dimensional gels electrophoresis was performed as previously described [54]. The protein separation pattern was visualized in 2-D gels by colloidal Coomassie Blue staining [55]. The gels were scanned in a GS-800 densitometer (Bio-Rad, Hercules, CA, USA). Digital images were analyzed and compared using the ImageMaster 2D Platinum 7.0 software (GE Healthcare). The densities of protein spots, which were normalized using the total densities of all valid and matched spots in a set of gels, were quantitatively compared between diabetic and normal control groups, using the pancreatic protein samples from three diabetic mice and three control mice. Data are presented as fold change (increase or decrease) *versus* matched controls. Each experiment was performed in triplicate. Statistical differences between groups were calculated using the Student’s *t*-test. Statistical significance was considered at *p* < 0.05.

### 3.3. Mass Spectrometry

Once the digital image of each gel was compared against the rest, the electrophoretic entities of interest were cut, reduced, alkylated, digested and automatically transferred to a MALDI analysis target by a Proteineer SP II and DP robot using the SPcontrol 3.1.48.0 v software (Bruker Daltonics, Bremen, Germany), with the aid of a DP Chemicals 96 gel digestion kit (Bruker Daltonics) and processed in a MALDI-TOF Autoflex (Bruker Daltonics) to obtain a mass fingerprint. We performed 100 satisfactory shots in 20 shotsteps, the peak resolution threshold was set at 1500, the signal/noise ratio of tolerance was 6, and contaminants were not excluded. The spectrum was annotated by the Flex Analysis 1.2 v SD1 Patch 2 (Bruker Daltonics). The search engine MASCOT [56] was used to compare the fingerprints against the UNIPROT [57] release 2010–09 database with the following parameters: Taxon-mouse, mass tolerance of up to 200 ppm, one miss-cleavage allowed, and as the fixed modification Carbamidomethyl cysteine and oxidation of methionine as the variable modification.

### 3.4. Bioinformatics Analysis

To understand the relationship among differentially expressed proteins in pancreas of *db*/*db* mice and their interaction with other proteins, we analyzed the interactome using STRING database 9.05 [47]. Analysis of differentially expressed proteins by Gene Ontology assignment was performed using DAVID v. 6.7 [48,58]. Proteins were uploaded into the DAVID functional annotation tool and compared to the mouse proteome background. Only enriched pathways and Gene Ontology Functional Annotation Tool terms with a minimum of 2-fold enrichment and a Fisher’s Exact test *p*-value ≤ 0.05 were considered, and the top Gene Ontology terms with protein change expression are reported as the proportion of genes involved relative to the total number of genes involved in the whole mouse genome background.

## 4. Conclusions

The animal model of type 2 diabetes mellitus used here the pathophysiology is comparable, if not equal to the disease observed in diabetic humans or in other animal models. Nevertheless, the differentially expressed proteins observed in the pancreas of *db*/*db* mice already suggest which are the mechanisms involved in the pathogenesis of diabetes, such as dysfunctional ER-dependent and carbohydrate metabolism processes, together with a chronic inflammation. So the results shown in this research could serve as targets for the design of new drugs for the treatment of diabetes.
